# Evolution of a global regulator: Lrp in four orders of γ-Proteobacteria

**DOI:** 10.1186/s12862-016-0685-1

**Published:** 2016-05-20

**Authors:** Yvette Unoarumhi, Robert M. Blumenthal, Jyl S. Matson

**Affiliations:** Department of Medical Microbiology and Immunology, University of Toledo College of Medicine and Life Sciences, Toledo, Ohio USA; Program in Bioinformatics and Proteomics/Genomics, University of Toledo College of Medicine and Life Sciences, Toledo, OH USA

**Keywords:** Transcription factors, Phylogenomics, Enterobacteriales, Vibrionales, Pasteurellales, Alteromonadales

## Abstract

**Background:**

Bacterial global regulators each regulate the expression of several hundred genes. In *Escherichia coli*, the top seven global regulators together control over half of all genes. Leucine-responsive regulatory protein (Lrp) is one of these top seven global regulators. Lrp orthologs are very widely distributed, among both Bacteria and Archaea. Surprisingly, even within the phylum γ-Proteobacteria (which includes *E. coli*), Lrp is a global regulator in some orders and a local regulator in others. This raises questions about the evolution of Lrp and, more broadly, of global regulators.

**Results:**

We examined Lrp sequences from four bacterial orders of the γ-Proteobacteria using phylogenetic and Logo analyses. The orders studied were Enterobacteriales and Vibrionales, in which Lrp plays a global role in tested species; Pasteurellales, in which Lrp is a local regulator in the tested species; and Alteromonadales, an order closely related to the other three but in which Lrp has not yet been studied. For comparison, we analyzed the Lrp paralog AsnC, which in all tested cases is a local regulator. The Lrp and AsnC phylogenetic clusters each divided, as expected, into subclusters representing the Enterobacteriales, Vibrionales, and Pasteuralles. However the Alteromonadales did not yield coherent clusters for either Lrp or AsnC. Logo analysis revealed signatures associated with globally- vs. locally- acting Lrp orthologs, providing testable hypotheses for which portions of Lrp are responsible for a global vs. local role. These candidate regions include both ends of the Lrp polypeptide but not, interestingly, the highly-conserved helix-turn-helix motif responsible for DNA sequence specificity.

**Conclusions:**

Lrp and AsnC have conserved sequence signatures that allow their unambiguous annotation, at least in γ-Proteobacteria. Among Lrp orthologs, specific residues correlated with global vs. local regulatory roles, and can now be tested to determine which are functionally relevant and which simply reflect divergence. In the Alteromonadales, it appears that there are different subgroups of Lrp orthologs, one of which may act globally while the other may act locally. These results suggest experiments to improve our understanding of the evolution of bacterial global regulators.

**Electronic supplementary material:**

The online version of this article (doi:10.1186/s12862-016-0685-1) contains supplementary material, which is available to authorized users.

## Background

Global regulators (GRs) are transcription factors that, collectively, play a critical role in bacteria: they help to coordinate the responses of the cell’s thousands of genes to complex environmental changes [1]. In contrast to local regulators, which each control transcription of a small number of genes, GRs each control hundreds of genes. The top seven GRs in *Escherichia coli* (ArcA, Crp, Fis, Fnr, Ihf, Lrp, and NarL) together control about half of all its genes [2]. While each GR may have a general functional role, the genes controlled by each GR (its regulon) can specify a variety of disparate functions [2–5].

**Fig. 1:**
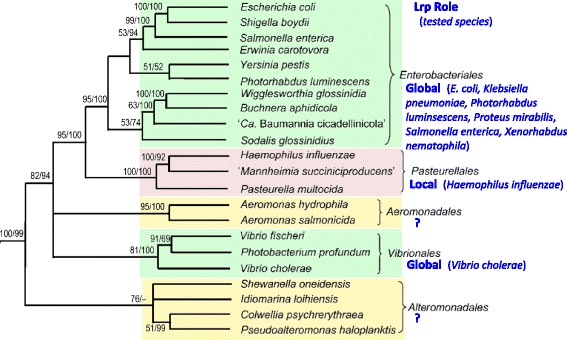
Role of Lrp superimposed on core genome phylogeny. Five orders of the γ-Proteobacteria are shown, adapted (with permission) from a maximum likelihood tree generated by Gao et al. [8], and based on the concatenated sequences of 36 highly-conserved proteins. They used both maximum parsimony (MP) and maximum likelihood (ML) approaches, and the two numbers are the proportion of the puzzling quartets (ML)/% bootstrap scores (MP) that supported the given node. For each order, the colored shading and text to the right indicates the role played by Lrp in tested species (*green* = global, *pink* = local), and the tested species are also indicated. For two orders, indicated by “?” and *yellow* shading, the role of Lrp has not yet, to our knowledge, been tested

Despite their importance, a number of fundamental questions about GRs remain unanswered, in particular regarding the evolution of their global roles (see [6, 7]). Here, we use Lrp as a model GR to begin to address the question of GR evolution, focusing on the phylum and class that includes *E. coli* – the γ-Proteobacteria. This choice was made in part because, within different members of that phylum, there are examples of Lrp playing local and global roles. Further, this difference in Lrp role does not follow the same phylogenetic pattern as the core genome (Fig. 1, adapted from [8]). Specifically, Lrp appears to play global roles in many species of the order Enterobacteriales [9–16]; and in at least one [13] and possibly a second [17] species in the Vibrionales. In contrast, Lrp plays a local role (control of branched-chain amino acid biosynthesis) in the one tested species in the Pasteurellales, *Haemophilus influenzae* [18]. However, the Pasteurellales core genomes appear to be more closely related to those of Enterobacteriales than either is to the core genomes of the Vibrionales. While the relationship between these bacterial orders (and Fig. 1) is derived from analysis of concatenated gene sequences, and thus have some level of uncertainty [19], it is nevertheless clear that Lrp plays different roles in closely-related bacterial orders, and for that reason is a good target for our studies on GR evolution.

Lrp has the functional flexibility one might expect of a GR. Lrp was originally named for its response to a coregulator (Leucine-responsive regulatory protein [20–22]), though subsequent analysis showed that it responds to a wider range of amino acids than just leucine [23]. Lrp was later recognized as belonging to a very large and ancient protein family (PF01037), with members in both the Bacteria and the Archaea [24–26]. This family is called the FFRPs, for Feast or Famine Regulatory Proteins, and the great majority includes two broad functional domains [27]. First, an amino-proximal helix-turn-helix DNA-binding domain, and second a coregulator response domain called RAM (Regulation of Amino acid Metabolism) [27, 28]. The DNA sequence specificity of the Lrp helix-turn-helix is, in some cases, modulated by a flexible amino-terminal tail [29]. The RAM domain links coregulator levels to multimerization state, as follows. Lrp forms dimers that, in turn, tetramerize to form octameric rings with the helix-turn-helix domains exposed on the outer edge [30]. The DNA presumably wraps around this ring and, at least in the best-studied Lrp protein (from *Escherichia coli*; subsequently referred to as EcoLrp), apparently causes the octameric ring to open [31]. In the absence of coregulator, two EcoLrp octamers stack like coins to form a hexadecamer [32] and possibly larger complexes [33]. Leucine-RAM interactions favor dissociation of these 16mers back to two 8mers [34]. There is indirect evidence that the 16mers (low coregulator level) have higher affinity for DNA, while the 8mer (high coregulator level) has greater ability to activate transcription [35, 36]. Thus Lrp exhibits considerable regulatory flexibility – at high-affinity operator sites on the DNA, the coregulator has little effect on repression and may increase the extent of activation (the 8mer remains bound but 16mer dissociation increases activation capacity); while at lower affinity operator sites the coregulator reduces both activation and repression.

To study the evolution of Lrp among γ-Proteobacteria, we focused on two questions. First, does the phylogeny of Lrp more closely follow its host’s core genome, or instead primarily reflect its global vs. local role? Second, are there any signature sequences associated with the global vs. local roles that might be used predictively during genome annotations? To address these questions, we examined the sequence changes in Lrp in four bacterial orders of the class γ-Proteobacteria. For comparison, we also studied a paralog of Lrp called AsnC, which consistently acts as a local regulator, in *E. coli* controlling its own gene and the downstream *asnA* gene (and another downstream gene post-transcriptionally) [37, 38]; as well as three housekeeping genes to reflect the core genomes (*rpoB*, *recA*, and 16S rRNA).

## Results and discussion

We examined the global regulator (GR) Lrp in the class γ-Proteobacteria, focusing on two orders in which Lrp acts globally (Enterobacteriales, Vibrionales), and one in which it acts locally (Pasteurellales; see Fig. 1). In addition, we included one order in which the role of Lrp is currently unknown (Alteromonadales); this order is relatively closely related to the other three being studied and, like Vibrionales, includes many free-living marine bacteria. We included only species for which the genome sequence included orthologs for all of the genes we studied: *lrp*, *asnC*, 16S rRNA, *rpoB*, and *recA* (Table 1).

**Table 1:** Species used and accession numbers for their genomes and target genes

Species (abbr)	Genome	16S rRNA	RpoB	Lrp	AsnC	RecA
Enterobacteriales						
* Citrobacter youngae* (Cyo)	GG730299.1*	AB273741	EFE05379.1	EFE08594.1	EFE06470.1	EFE06891.1
* Dickeya dadantii* (Dda)	CP002038.1	AF520707	WP_038900031.1	ADM98245.1	WP_038921215.1	WP_013319098.1
* Escherichia coli* (Eco)	NC_000913.3	NR_102804.1	NP_418414.1	NP_415409.1	NP_418199.1	AIZ90260.1
* Klebsiella pneumonia*e (Kpn)	APVW01000146.1*	AF394537	CDQ52107.1	EOZ13501.1	B5XZL3_KLEP3	KFJ75935.1
* Photorhabdus asymbiotica* (Pas)	FM162591.1	NR_029093.1	CAQ82443.1	CAQ84933.1	KGM25900.1	CAQ85300.1
* Proteus mirabilis* (Pmi)	Y10417.1*	KM099410.1	KGA91942.1	CAA71443.1	B4F0D6_PROMH	KGY45908.1
* Salmonella enterica* Typhimurium (Sty)	NC_003197.1	DQ344537	AAF33499.1	NP_459935.1	NP_462775.1	NP_461750.1
* Serratia marcescens* (Sma)	U02276.1*	AF124036	KHO40608.1	AAA75466.1	KMJ03309.1	WP_015670993.1
* Yersinia enterocolitica* (Yen)	CP009367.1	NR_074308.1	AJJ28227.1	AJI83973.1	AHM76528.1	AJJ25926.1
* Yersinia pestis* (Ype)	AL590842.1	NR_074199.1	CAL22334.1	CAL20027.1	YP_002345098.1	CAL21898.1
Vibrionales						
* Grimontia indica* (Gin)	NZ_ANFM02000022.1*	FJ943235	EOD77459.1	WP_002539165.1	WP_002541578.1	WP_002537588.1
* Photobacterium aphoticum* (Pap)	BBMN01000005.1*	X74685	Q6LLW2.1	GAL04870.1	GAL07747.1	GAL08267.1
* Vibrio anguillarium* (Van)	CP002284.1	X71817	CDQ49128.1	AEH33539.1	CDQ51588.1	P26348.1
* Vibrio campbelli* (Vca)	JSFE01000003.1*	DQ980029	A7MXF1.1	KGR35110.1	KGR34405.1	WP_045384581.1
* Vibrio cholerae* N16961(Vch)	AE003852.1	AE004119	Q9KV30.2	AAF95052.1	NP_229730.1	P45383.1
* Vibrio fischeri* (Vfi)	NC_006840.2	CP000020	YP_205797.2	YP_204287.1	YP_203464.1	YP_203918.1
* Vibrio icthyoenteri* (Vic)	AFWF01000031.1*	HM771339	EGU40820.1	EGU46924.1	EGU37343.1	EGU37265.1
* Vibrio splendidus* (Vsp)	CH724173.1*	AJ515230	WP_029224582.1	EAP92110.1	EAP95563.1	WP_029225094.1
* Vibrio variabilis* (Vva)	JRWM01000006.1*	GU929924	KHA59154.1	KHA61343.1	GAL25042.1	GAL30153.1
* Vibrio vulnificus* (Vvu)	BA000037.2	BA000037	KFK71115.1	BAC94085.1	NP_932862.1	AIL71594.1
Pasteurellales						
* Actinobacillus succinogenes* (Asu)	CP000746.1	NR_074818.1	A6VKC5.1	ABR74465.1	WP_012072259.1	WP_011978916.1
* Aggregatibacter actinomycetem-comitans* (Aac)	NZ_CP007502.1	CP003496	EKX96954.1	WP_005540269.1	WP_005548336.1	Q9JRP9.1
* Avibacterium paragallinarum* (Apa)	NZ_AFFP02000001.1*	AY498868	KKB02504.1	WP_017806582.1	WP_017806647.1	KKB01216.1
* Chelonobacter oris* (Cor)	NZ_JSUM01000003.1*	EU331064	KGQ69613.1	WP_034612909.1	WP_034615444.1	KGQ69536.1
* Gallibacterium genomo* (Gge)	NZ_JPXX01000021.1*	AF228015	WP_039172822.1	WP_039173617.1	WP_013745649.1	KGQ37099.1
* Haemophilus ducreyi* (Hdu)	NC_002940.2	M63900	Q7VKL7.1	WP_010945324.1	WP_041603575.1	AAP95375.1
* Haemophilus Influenzae* (Hin)	JFZK01000018.1	M35019	AJO91604.1	KAI97579.1	WP_005649540.1	AJO91526.1
* Mannheimia haemolytica* (Mha)	NZ_CP006957.1	NR_102832.1	AKA12987.1	WP_006251058.1	WP_006248981.1	AAD53288.1
* Necropsobacter rosorum* (Nro)	NZ_CCMQ01000006.1*	NR_114428.1	WP_032093555.1	WP_032092886.1	WP_032093497.1	WP_032093315.1
* Pasteurella multocida* (Pmu)	NZ_CP008918.1	NR_103916.1	AAK03821.1	WP_005721107.1	WP_005718316.1	P95526.1
Alteromonadales						
* Ferrimonas futtsuensis* (Ffu)	NZ_KE383896.1	AB245515	WP_028110712.1	WP_028108266.1	WP_028110962.1	WP_028109920.1
* Ferrimonas senticii* (Fse)	NZ_AUGM01000029	DQ778094	WP_028117235.1	WP_028115272.1	WP_028117739.1	WP_028117049.1
* Idiomarina* sp. A28L (Isp)	AFPO01000011.1*	FJ404759	EGN75162.1	EGN75753.1	WP_007420625.1	EGN75701.1
* Moritella dasanensis* (Mda)	NZ_AKXQ01000041.1*	EF192283.1	WP_017222504.1	WP_017223704.1	WP_017222470.1	WP_017221889.1
* Pseudoalteromonas luteoviolacea* (Plu)	NZ_AUSV01000032.1*	X82144	KID54553.1	WP_023398863.1	WP_023399751.1	ESP93629.1
* Pseudoalteromonas tunicata* (Ptu)	NZ_CH959301.1*	DQ005908	EAR26370.1	WP_009839786.1	WP_009840643.1	WP_009837615.1
* Psychromonas aquimarina* (Paq)	NC_008709.1	AB304805	WP_028863208.1	WP_011771837.1	WP_028864437.1	WP_028864512.1
* Shewanella frigidimarina* (Sfr)	NC_008345.1	NR_074814.1	WP_011635633	WP_011637260.1	WP_011636002.1	Q086A0.1
* Shewanella loihica* (Slo)	CP000606.1	NR_074815.1	ABO22023.1	ABO23891.1	WP_014609941.1	ABO23085.1
* Shewanella pealeana* (Spe)	NC_009901.1	NR_074821.1	A8GYW9.1	WP_012155470.1	WP_012154000.1	ABV86522.1

*contigs and/or full genome were not available at time of writing

### Phylogeny and identifying motifs of the paralogs Lrp and AsnC

**Fig. 2:**
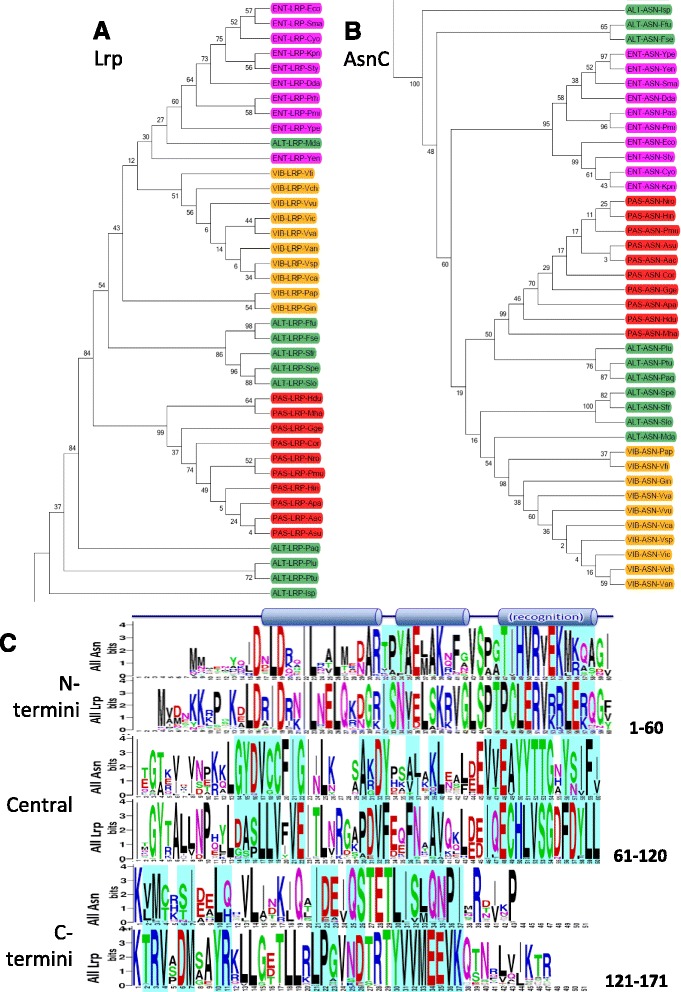
Phylogeny and comparison of the paralogs Lrp and AsnC. Maximum likelihood phylogeny was constructed using the **a** Lrp and **b** AsnC protein sequences. The numbers above or below the internal branches show bootstrap values (%). Color keys indicate the different orders: *magenta* = Enterobacteriales (Ent), *orange* = Vibrionales (Vib), *green* = Alteromandales (Alt), *red* = Pasteurellales (Pas). **c** Logo comparison of all 40 Lrp vs. all 40 AsnC sequences. Areas visually identified as showing conserved differences are shaded in *cyan*

We aligned the 80 amino acid sequences (40 Lrp and 40 AsnC, with both Lrp and AsnC sequences coming from the same genomes), and then subjected them to phylogenetic analysis (see Methods). The Lrp and AsnC sequences clustered separately, as shown in Fig. 2a, b, and Additional file 1: Figure S1 (which shows the original joined Lrp/AsnC tree). This is not surprising, but requires a clarification. Namely, there were several cases of generic or mis-annotation associated with the genome sequences, where both genes were called “AsnC family” or something similar. We used logo analysis, which reveals patterns and extents of conservation within a set of orthologs [39, 40]. This analysis revealed both universally-conserved residues (within all Lrp + AsnC sequences), and residues that were highly conserved but distinct between Lrp and AsnC (indicated by shading in Fig. 2c). These differences were then used to assign “AsnC family” polypeptides to the correct category. [Note that, unless otherwise specified, residue numbers refer to the mass alignment positions, and these may differ from the numbering in the individual GenBank records.]

To assess the diagnostic value of these conserved sequence differences, we used the longest Lrp-specific segment (106-IQECHLVSGdFDYLLkTRV-124, where the two lower case symbols are not unique to Lrp; see Fig. 2c) in a BLASTP search against the full nonredundant GenBank dataset. We examined the first 250 hits that had 100 % query coverage and 100 % identity. Of these, 64 % were annotated as Lrp, 30 % as “AsnC family”, 3 % as “hypothetical protein”, and <1 % each as “putative Lrp”, “transcriptional regulator” or “putative transcriptional regulator”. There were two cases, both in Vibrio genomes, annotated as the proline utilization regulator PutR. Significantly, there were no cases annotated as being AsnC. Conversely, when we used the equivalent segment from the AsnC sequence (VVEAYYTTG*YSIFIk*M; * = wildcard), there were no cases annotated as “Lrp” – the great majority were labeled “transcriptional regulator”, with 8 % annotated as “AsnC family” and 6 % as AsnC. The sequence segments highlighted in Fig. 2c may thus be useful in properly annotating Lrp and AsnC proteins, at least within the γ-Proteobacteria.

### Unusual phylogenies associated with Alteromonadales

**Fig. 3:**
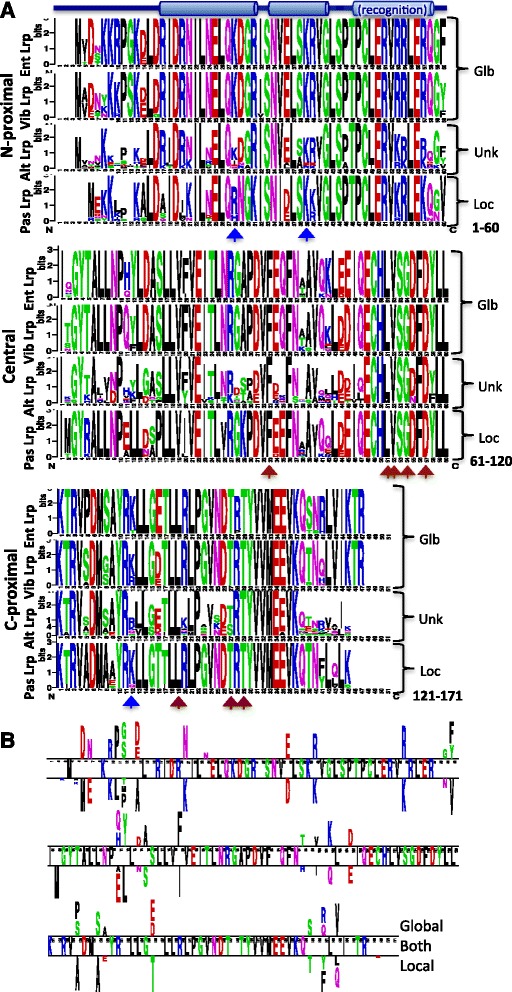
Comparison of Lrp orthologs grouped by order. **a** The ten Lrp sequences from each order were used to generate aligned Logos, in order to compare globally- (Glb at right) and locally-acting (Loc) orthologs. The orders are abbreviated: Ent = Enterobacteriales, Vib = Vibrionales, Alt = Alteromonadales, Pas = Pasteurellales. The vertical arrows indicate positions of lysine acetylation (*blue*, from [47]) or formation of the coregulator binding pocket (*red*). See text for details. **b** Two-sample Logo comparing the global (Ent + Vib) and local (Pas) Lrp orthologs. Letters between the lines indicate amino acid residues that are conserved in both sets, symbols above the lines are selectively enriched in the globally-acting Lrp set, and symbols below the lines are selectively enriched in the locally-acting Lrp set

Closer examination of the Lrp and AsnC phylogenetic clusters reveals that the sequences cluster as expected by order for the Enterobacteriales, Vibrionales and Pasteurellales (Fig. 2 parts a and b). However the Alteromonadales do not yield a single cluster for either protein (green shading), and this is true even when branches having <70 % bootstrap support are collapsed (Additional file 1: Figure S1B). This is consistent with the order-specific logos we generated for Lrp (each derived from the 10 species used from each order), shown in Fig. 3a. There are a number of positions at which the Alteromonadales logo shows substantially lower conservation than in the other three orders. An example is in the carboxy-proximal region (bottom of figure), positions 143-146, which is a strongly conserved GVND in three orders, and much more variable among the Alteromonadales. The differences between the two Alteromonadales clusters are shown, for both Lrp and AsnC, in Additional file 1: Figure S2. We used two-sample Logo analysis [41], and the results reveal significant subcluster-specific sequence differences distributed over the entire length of the polypeptides. The subclusters thus reflect substantial sequence differences, not seen among Lrp or AsnC orthologs from the other three orders.

**Fig. 4:**
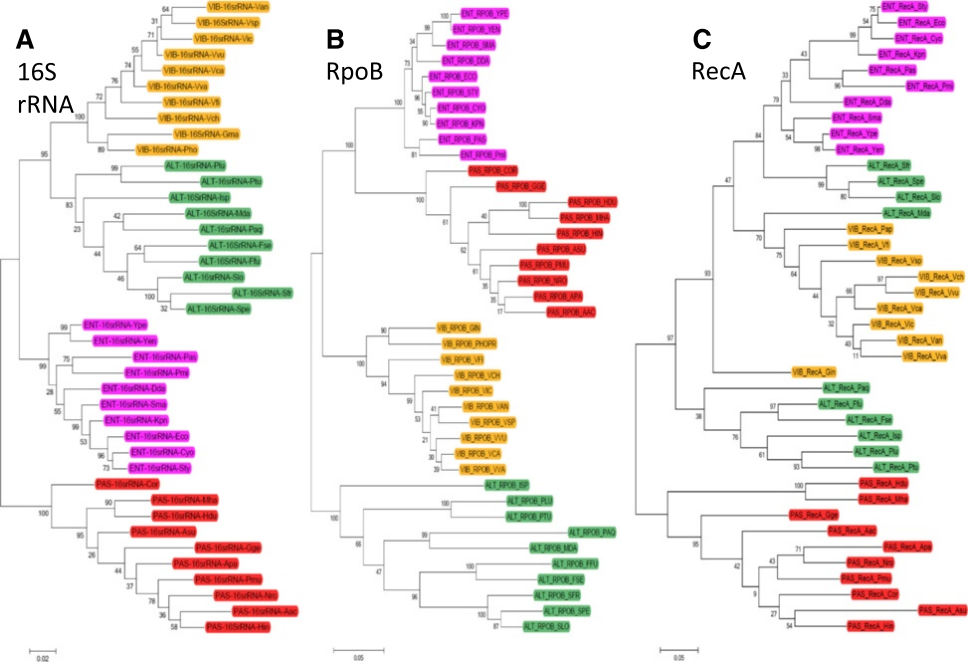
Phylogeny of conserved housekeeping genes. Maximum likelihood phylogeny constructed for **a** 16S rRNA, **b** RpoB, and **c** RecA from the four bacterial orders. Colors are as assigned for Fig. 1

We considered the possibility that the core genomes for the Alteromonadales species we chose were inconsistently assigned. However, the phylogenies for two highly-conserved genes (16S rRNA, and RpoB – a large subunit of RNA polymerase) cluster as expected for all four orders (Fig. 4, parts a and b). On the other hand, a third conserved gene – RecA – shows Alteromonadales-specific split clustering as was seen for Lrp and AsnC (Fig. 4c). The order-specific logos for RecA, unlike the case for Lrp, do not reveal specific regions in which the Alteromonadales have unusual sequence variability (Additional file 1: Figure S3).

Some bootstrap values in Fig. 1 are relatively low, particularly in the AsnC tree, but the separation of Paq, Plu, Ptu, and Isp Lrp orthologs from the other Alteromonadales Lrps is robust even when low-support nodes are collapsed (Additional file 1: Figure S1B). The separation of the two Alteromonadales RecA clusters also appears to be robust (Fig. 4c). Comparing the Alteromonadales Lrp, AsnC and RecA subclusters, there are some consistencies (Ffu/Fsp, Paq/Plu/Ptu, and Spe/Sfr/Slo are always together with one another) and some differences (e.g., Mda and Isp have more variable associations). Detailed exploration of this phylogenetic pattern is beyond the scope of this study, but we note that similar disparities have been seen in some other studies that include Alteromonadales (e.g., MntX Mg^++^ transporter, Fig. S3a in [42]; various genes in [43]). This might reflect recent divergences or active horizontal gene transfer.

### Potential differences between globally- and locally-acting Lrp orthologs

Even changing 1-2 amino acids in a transcription factor can significantly modify its regulatory activity [44, 45]. One of our major goals for this study was to identify sequence signatures that might be associated with global- vs. local-regulatory roles for Lrp. Accordingly, we used two-sample logo analysis [41] to compare the 20 presumed globally-acting Lrps (Enterobacteriales + Vibrionales) to the 10 presumed locally-acting Lrp orthologs (Pasteurellales) (Fig. 3b). While bearing in mind the caveat that the number of genes controlled by Lrp has been tested directly in few of the 30 species included in this analysis, the residues identified by this analysis are testable candidate contributors to the global or local functionality of Lrp.

We consider the differing residues in four groups. First is the N-terminal 21 residues. This includes an N-terminal tail that plays a role in DNA binding [31] and sequence specificity (at least in Lrp from *E. coli*, *P. mirabilis*, and *V. cholerae*; [29]). The Pasteurellales Lrps have shorter and more variable N-termini. The two-sample Logo shows seven substantial differences in this region, including four differences over five residues, from positions 10-14.

Second is residues 36-60, which includes the DNA-recognizing helix-turn-helix (HTH) domain. Four major differences distinguish the globally- and locally-acting Lrp orthologs in this region. All four are relatively conservative, with one Glu/Asp difference, two Arg/Lys, and one Phe/Val. However the D/E and one R/K change is within the first HTH helix, another R/K is within the recognition helix, and the F/V is three residues after the recognition helix. Between these and the differences in the N-terminal region, it is possible that sequence specificity differs between these two groups.

Third is residues 61-135, which includes the coregulator-binding RAM domain. There are nine residues with substantially-conserved differences between the global and local Lrp sets. None of the changes directly involve residues that form the coregulator-binding pocket (red arrows in Fig. 3a). Three of the changes result in charge differences; two involve shifts from an aromatic (global) to a branched (local) sidechain (Tyr/Leu and Phe/Ile).

Finally, there are substantial differences at the C-termini, residues 159-171. At least in *E. coli* Lrp, this region is associated with changes in multimerization in response to the coregulator leucine [32]. In the Enterobacteriales and Vibrionales, this is a highly-conserved LVIKTR motif, while in the Pasteurellales, only the K of that motif is (partially) conserved (Fig. 3a). The two-source Logo shows three particularly significant conserved differences, of which the central one is most stark – Arg or Gln in the global Lrp set vs. Tyr or Phe in the local Lrp set (Fig. 3b).

**Fig. 5:**
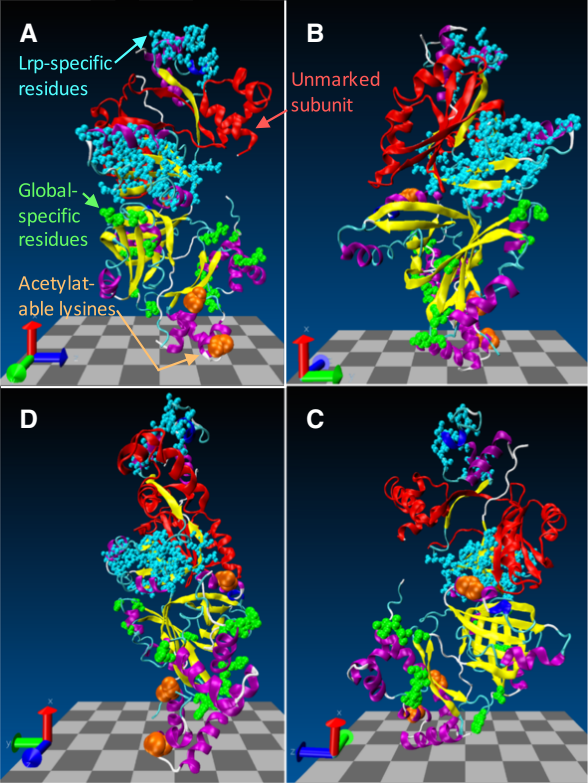
Visualization of residues of interest in context of Lrp 3D structure. The program VMD 1.9.2 was used to visualize half of an octameric ring of *E. coli* Lrp subunits (from PDB 2GQQ). VMD is developed with NIH support by the Theoretical and Computational Biophysics group at the Beckman Institute, University of Illinois at Urbana-Champaign. **a**-**d** are successive 90° rotations about the vertical axis. The topmost subunit has *cyan spheres* highlighting residues associated with Lrp-specific signatures (see Fig. 2c), the next subunit is shown in *red* without additional highlighting, the next subunit shows in *orange spheres* the lysines that can be acetylated (see Fig. 3a), and the bottom subunit shows in *green spheres* the residues associated with globally-acting Lrp orthologs (see Additional file 1: Table S1 for position numbers of all highlighted residues)

Figure 5 shows the distribution of these candidate role-specifying residues in the context of the Lrp three-dimensional structure. The figure shows four *E. coli* Lrp subunits (half of an octamer), with one subunit all in red to illustrate its overall shape, and another subunit having candidate role-specifying residues as green spheres; as indicated in Fig. 3, these are distributed over the full length of the protein (position numbers are given in Additional file 1: Table S1). At least some of these apparent local vs. global differences, of course, may simply reflect genetic drift. But they represent a set of targets for specific functional testing in attempts to understand the differences between globally- and locally-regulating Lrp orthologs, and the more general question of what distinguishes these two classes of regulators.

### Lysine acetylation

Another potentially important level of control for GRs is post-translational modification. *E. coli* has enzymes that generate or remove acetyl groups from lysine residues [46]. While the role of Lrp acetylation has not been studied directly, a whole-proteome analysis of *E. coli* revealed that Lrp is substantially acetylated on three lysines: K28, K39 and K132 (supplementary data in [47]). These positions are indicated by blue arrows in Fig. 3a (where the numbering reflects the multiple alignment), and orange spheres in Fig. 5. K132 is less-well conserved in Vibrionales than in the other two orders, but is not strongly conserved in any of the orders. K39 is conserved in both the global and local Lrp sets, and is within the upstream helix of the HTH motif where acetylation might interfere with formation of a salt bridge to the DNA backbone, or even promote DNA binding [48]. Interestingly, K28 is strongly conserved in the Enterobacteriales and Vibrionales (global), but is replaced by Arg or His in the Pasteurellales (local), preserving the positive charge but not the acetylation potential. It seems important to explore in future the possible role of Lrp acetylation, especially in bacteria where Lrp plays a global role.

### What is the likely role of Lrp in the Alteromonadales?

From the analyses presented in this study, it might be possible to make a testable predication as to the role (global or local) of Lrp in the Alteromonadales. From the phylogenetic relationships shown in Fig. 2a, it seems possible that Lrp might play different roles in different species, corresponding to the distinct subclusters. However, the bootstrap values make it difficult to clearly assign any Lrp cluster as being particularly closely associated with the Pasteurellales (local role). Figure 3 suggests that at least the majority of Lrp orthologs in the Alteromonadales play a local role, based in particular on the missing or degenerate N-terminal and C-terminal regions. On the other hand, regarding some of the specific differences between local and global Lrp ortholog sets shown in Fig. 3b, the Alteromonadales more closely resemble the global set. For example, in the Alteromonadales Lrp set Asp14 is more common than Ala14 (which we notate as D14 > A), along with N21 > K, E36 > D, R40 > K, F60 > V, F80 > V, S128 > A, and D/E136 > T. Only one of these positions, residue 21, differs substantially between the Alteromonadales Lrp subclusters (Additional file 1: Figure S2).

These results are all ambiguous and make prediction difficult, but they are the result of comparing combined sequences. We therefore aligned *Moritella dasanensis* (Mda) Lrp individually to the known global regulator *E. coli* Lrp (Additional file 1: Figure S4), based on Mda’s outlying position among the Enterobacteriales in the phylogenetic analysis shown in Fig. 2a. These two Lrp orthologs share 91 % identity, and it is particularly striking that the conserved N- and C-terminal sequences characteristic of the global forms of Lrp are conserved in Mda, even though they are missing from most Alteromonadales Lrp orthologs. Also, 8/8 global signature residues (see preceding paragraph, underlined in Additional file 1: Figure S4) are identical in Eco and Mda Lrp. It therefore seems reasonable to predict that Lrp will be found to play a global role in *M. dasanensis*. At the other extreme (Fig. 2a) is the Lrp ortholog from *Idiomarina spp.* (Isp). It has just 68 % identity to EcoLrp, comparable to the Isp identity with the known local regulator from *Haemophilus influenzae* (Hin), and matches Eco at just 2/8 signature residues. Thus it seems more likely that in Isp Lrp would be found to play a local regulatory role.

In contrast to the Alteromonadales, the Lrp orthologs we studied in the other three orders appear likely to play consistent roles – all local in Pasteurellales; all global in the Enterobacteriales and Vibrionales. Changes in bacterial regulatory networks, due in part to horizontal gene transfer, is well documented [7, 49]. It remains to be determined experimentally whether the proposed global/local role variation among Alteromonadales Lrp orthologs is real, but it raises questions about how the bacteria adapted to the gain or loss of a GR that would presumably have occurred during their evolution. Regarding loss, in *E. coli* deletion of the gene for Lrp does not greatly affect growth in rich media, but has profound effects under some conditions, and makes the cells far more sensitive to mutations affecting other regulators [50, 51]. Regarding displacement, in *E. coli* exchanging one Lrp ortholog for another (*Vibrio cholerae* or *Proteus mirabilis* for *E. coli*) results in only partial retention of the normal regulation of the several hundred genes in the Lrp regulon, despite their identical HTH motifs [13]. Introducing a new GR where none existed before would probably be the least disruptive of these scenarios, allowing new genes to join the regulatory network over time. Presumably this latter gain-of-function scenario would result in substantially different regulon memberships than might be expected from simple species divergence, and this might provide additional evidence for past importation of a GR gene.

## Conclusions

The global regulator Lrp, and its locally-acting paralog AsnC, have conserved sequence signatures that allow their unambiguous annotation, at least in γ-Proteobacteria. Among Lrp orthologs, we identified residues correlated with global vs. local regulatory roles, that can guide future experiments to determine which of them are functionally significant and which reflect simple divergence. Based on these observations, it was possible to make reasoned predictions for the global vs. local role of Lrp in the Alteromonadales, a bacterial order in which the role of Lrp has not yet been determined. Unlike the other three orders we studied here, it appears that in the Alteromonadales there are different subgroups of Lrp orthologs, one of which may act globally while the other may act locally. Together, these results suggest defined experimental avenues to improve our limited understanding of the evolution of global regulatory transcription factors in bacteria.

## Methods

### Sequence retrieval

Sequences were retrieved from the NCBI database (http://www.ncbi.nlm.nih.gov/). From each of the four orders we studied, we chose ten species having a known genome sequence that included orthologs for both Lrp and – for comparison – its locally-acting paralog AsnC and the core genome housekeeping genes 16S rRNA, *rpoB*, and *recA*. The species were chosen to, as far as possible, broadly represent the genera in each order. Table 1 shows the species used, along with accession numbers for the genome sequences and studied genes.

### Phylogenetic analyses

Multiple alignments of protein sequences were generated using MUSCLE and CLUSTALΩ [52, 53] with default parameters. Maximum likelihood phylogeny was constructed using the multiple sequence alignment results in FASTA format using the best parameters for the presented dataset by MEGA software (v6) (www.megasoftware.net/) [54]. Distance estimations were obtained by the pre-imputed JTT amino-acid substitution model [55] with 1000 bootstrap simulations. MEGA can use either the Dayhoff/PAM or JTT substitution matrices, and the JTT modeling was found to be optimal for the purpose of this study.

### Logo analyses

We used WebLogo (weblogo.berkeley.edu) to determine extent of conservation in aligned sequence sets [39, 40], and two-sample Logo (www.twosamplelogo.org) to compare two sets of aligned sequences [41].

## Additional file

Additional file 1: Figures S1-S4. The figures show a universal phylogeny of 80 Lrp and AsnC sequences, two-source Logos comparing Alteromonadales subclusters for Lrp and AsnC, order-specific Logos for RecA, and alignments of selected individual Lrp sequences. Table S1. The table shows residues chosen for highlighting in the structural representation of Lrp shown in Fig. 5. (PDF 2483 kb)

## Abbreviations

FFRP: feast-or-famine regulatory protein
GR: global regulatory transcription factor
HTH: helix-turn-helix DNA-binding motif
Lrp: leucine-responsive regulatory protein
RAM: regulation of amino acid metabolism motif
