# Supplementary material for: Evolution of a global regulator: Lrp in four orders of γ-Proteobacteria
Source: BMC Evol Biol. 2016 May 20;16:111. doi: 10.1186/s12862-016-0685-1 (PMC4875751; doi:10.1186/s12862-016-0685-1)
Supplement: Additional file 1: Figures S1-S4. — The figures show a universal phylogeny of 80 Lrp and AsnC sequences, two-source Logos comparing Alteromonadales subclusters for Lrp and AsnC, order-specific Logos for RecA, and alignments of selected individual Lrp sequences. Table S1. The table shows residues chosen for highlighting in the structural representation of Lrp shown in Fig. 5. (PDF 2483 kb) [file 12862_2016_685_MOESM1_ESM.pdf]

Supplementary Information for

Evolution of a Global Regulator: Lrp in Four Orders of γ-Proteobacteria

Yvette Unoarumhi, Robert M. Blumenthal and Jyl S. Matson*

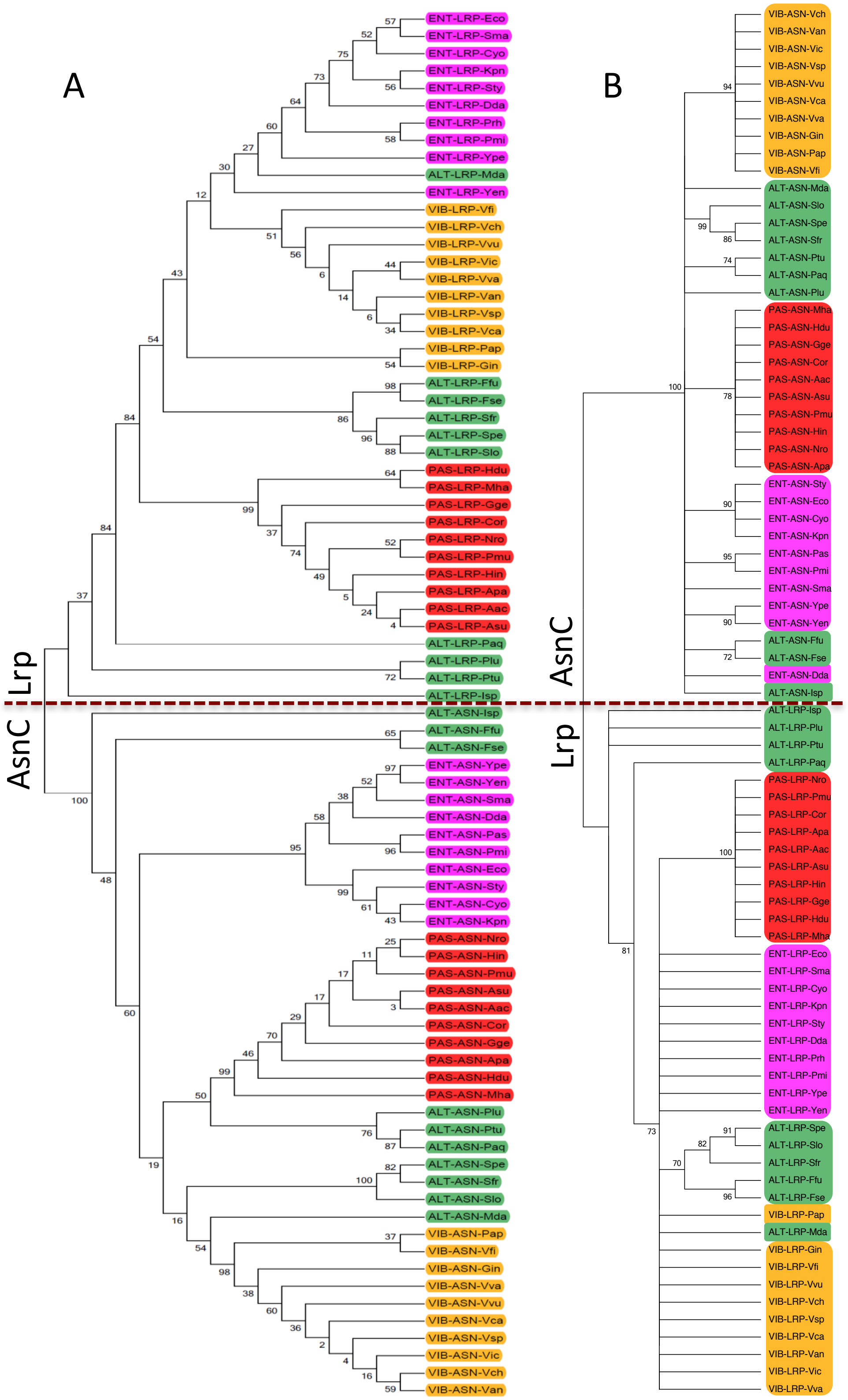

Figure S1. Phylogenetic analysis of 40 paired Lrp and AsnC sequences from four orders of γ-Proteobacteria. Maximum likelihood phylogeny was constructed using Lrp and AsnC protein sequences. The numbers above or below the internal branches show bootstrap values (%). Color keys indicate the different orders: magenta = Enterobacteriales (Ent), orange = Vibrionales (Vib), green = Alteromandales (Alt), red = Pasteurellales (Pas). **A** – Full tree. **B** – Tree with nodes collapsed if they had <70% bootstrap support.

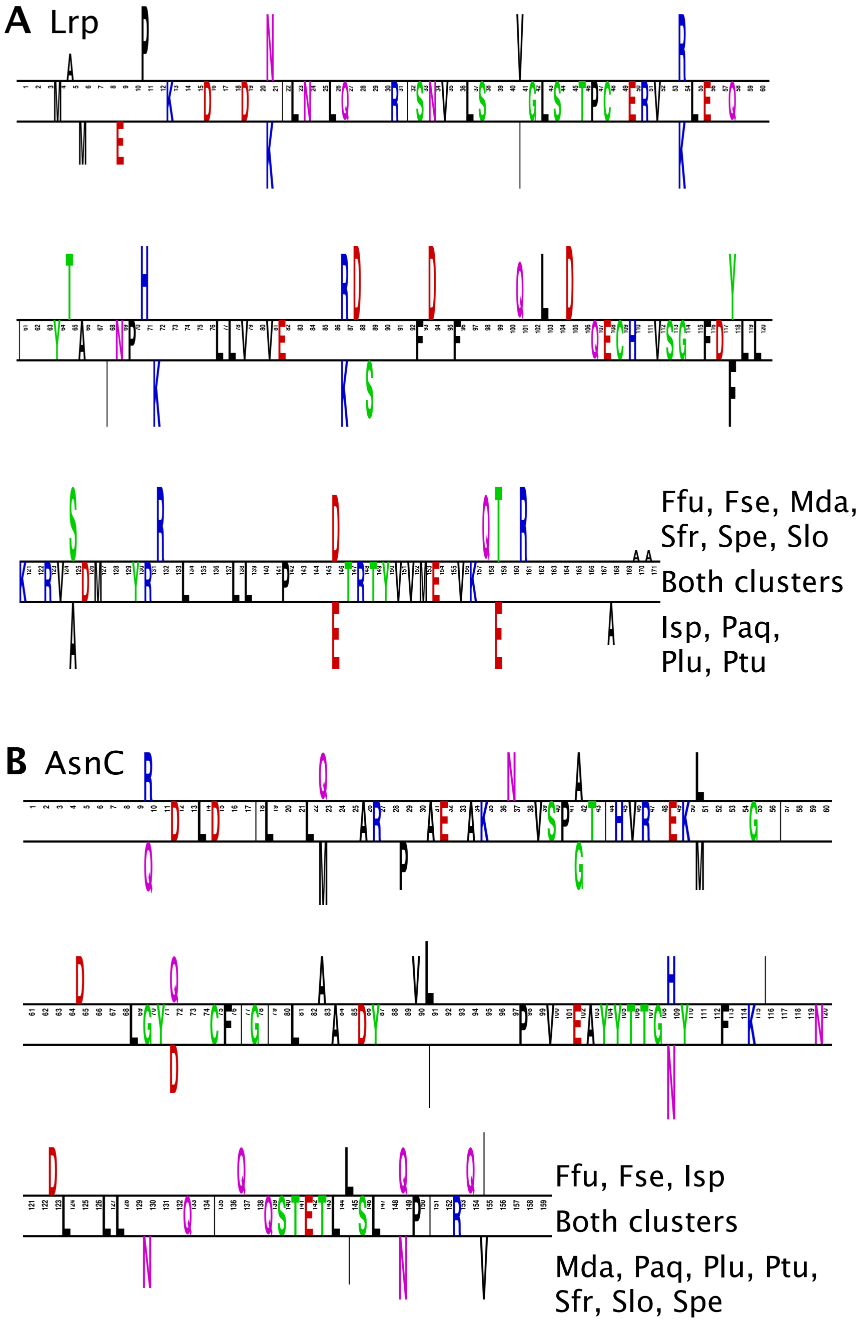

Figure S2. Comparison of orthologs from separate phylogenetic clusters in Alteromonadales. Two-sample Logo analysis was used to compare the orthologs of Lrp (**A**) or AsnC (**B**) from separate phylogenetic clusters (see Figure S1). The species abbreviations are listed in Table 1.

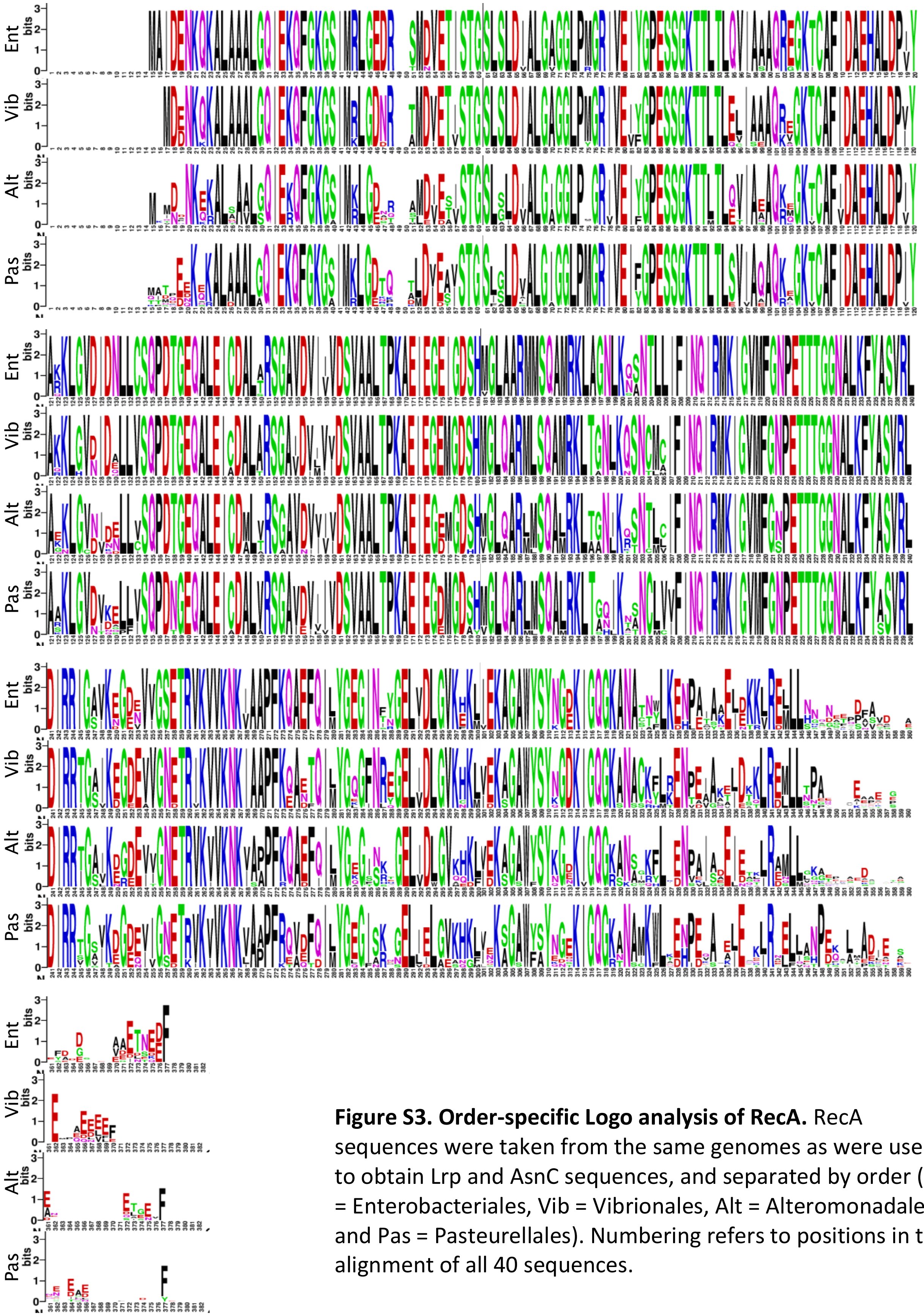

Figure S3. Order-specific Logo analysis of RecA. RecA sequences were taken from the same genomes as were used to obtain Lrp and AsnC sequences, and separated by order (Ent = Enterobacteriales, Vib = Vibrionales, Alt = Alteromonadales, and Pas = Pasteurellales). Numbering refers to positions in the alignment of all 40 sequences.

```
A.  Score          Expect        Identities      Positives       Gaps
303 bits(777)  2e-102         149/164(91%)  156/164(95%)  0/164(0%)

Mda      1     MMEVKTRPMKELDRIDRNILNELQKDGRISNVELSKRVGLSPTPCLERVRRLERQGYITG  60
               M++ K RP K+LDRIDRNILNELQKDGRISNVELSKRVGLSPTPCLERVRRLERQG+I G
Eco      1     MVDSKKRPGKDLDRIDRNILNELQKDGRISNVELSKRVGLSPTPCLERVRRLERQGFIQG  60

Mda     61     YTAILNPQFLDASLLVFVEITLNRGAADVFEQFNKAVQELEEIQECHLVSGDFDYLLKTR  120
               YTA+LNP FLDASLLVFVEITLNRGA DVFEQFN AVQ+LEEIQECHLVSGDFDYLLKTR
Eco     61     YTALLNPHFLDASLLVFVEITLNRGAPDVFEQFNTAVQKLEEIQECHLVSGDFDYLLKTR  120

Mda    121     VCDMSAYRRLLGETLLRLPGVNDTRTYVVMEEVKQSNRLVIKTR  164
               V DMSAYR+LLGETLLRLPGVNDTRTYVVMEEVKQSNRLVIKTR
Eco    121     VPDMSAYRKLLGETLLRLPGVNDTRTYVVMEEVKQSNRLVIKTR  164


B.  Score          Expect        Identities      Positives       Gaps
213 bits(542)  6e-67          102/151(68%)  125/151(82%)  0/151(0%)

Isp      9     IDRLDRKILRILQERGRISNVLLAKEVGLSPTPCLERVRKLERNGVIKGYHARIDPDKLG  68
               +DR+DR IL  LQ+ GRISNV L+K VGLSPTPCLERVR+LER GVI+GY A ++P  L
Eco     12     LDRIDRNILNELQKDGRISNVELSKRVGLSPTPCLERVRRLERQGFIQGYTALLNPHYLD  71

Isp     69     TPLLVFVEITLSKTSSDVFAEFSDAARKTDEILECHLVSGDFDFLLKARVADMAAYRKLL  128
                 LLVFVEITL++ + DVF +F+ A +K +EI ECHLVSGDFD+LLK RV DM+AYRKLL
Eco     72     ASLLVFVEITLNRGAPDVFEQFNTAVQKLEEIQECHLVSGDFDYLLKTRVPDMSAYRKLL  131

Isp    129     GETLLNMPGVNESRTYVVMEAVKQENKVVIK  159
               GETLL +PGVN++RTYVVME VKQ N++VIK
Eco    132     GETLLRLPGVNDTRTYVVMEEVKQSNRLVIK  162


C.  Score          Expect        Identities      Positives       Gaps
201 bits(510)  2e-65          98/152(64%)   120/152(78%)  0/152(0%)

Isp      8     TIDRLDRKILRILQERGRISNVLLAKEVGLSPTPCLERVRKLERNGVIKGYHARIDPDKL  67
                +D +D KIL  LQ  G+ISN+ L+K+VGLSPTPCLERV++LE+ GVI GY A ++P+ L
Hin      8     ALDAIDIKILNELQRNGKISNIDLSKKVGLSPTPCLERVKRLEKQGVIMGYRALLNPELL  67

Isp     68     GTPLLVFVEITLSKTSSDVFAEFSDAARKTDEILECHLVSGDFDFLLKARVADMAAYRKL  127
                 PLLV VEITL +   DVF EF+ A ++ DEI ECHLVSGDFD+LLK RVADMA YRKL
Hin     68     DAPLLVIVEITLVRGKPDVFEEFNAAIQELDEIQECHLVSGDFDYLLKTRVADMAEYRKL  127

Isp    128     LGETLLNMPGVNESRTYVVMEAVKQENKVVIK  159
               LG TLL +PGVN++RTYVVME VKQ N +V+K
Hin    128     LGSTLLRLPGVNDTRTYVVMEEVKQTNYLVLK  159
```

Figure S4. Single-sequence alignments of divergent Alteromonadales Lrp orthologs to sequences of Lrps playing known global or local roles. (**A**) Alignment of *Moritella dasanensis* (Mda) to *E. coli* (Eco, global) Lrp. (**B**) Alignment of *Idiomarina spp.* (Isp) to Eco Lrp. (**C**) Alignment of Isp to *Haemophilus influenzae* (Hin, local) Lrp.

Table S1. Positions of *E. coli* Lrp residues highlighted in Figure 5

Residues[a] distinguishing all Lrps from all AsnCs
ILE 29
SER 30
ASN 31
VAL 32
THR 43
PRO 44
CYS 45
LEU 46
GLU 47
ARG 50
ARG 51
LEU 52
GLU 53
ARG 54
GLN 55
ASP 71
ALA 72
SER 73
LEU 74
LEU 75
VAL 76
VAL 78
GLU 79
PRO 87
ASP 88
VAL 89
PHE 90
PHE 93
ASN 94
ALA 96
VAL 97
ILE 103
GLN 104
GLU 105
CYS 106
HIS 107
LEU 108
VAL 109
SER 110
GLY 111
PHE 113
ASP 114
TYR 115
LEU 116
LEU 117
THR 119
ARG 120
VAL 121
ASP 123
MET 124
TYR 127
ARG 128
LEU 138
PRO 139
GLY 140
ASN 142
ASP 143
TYR 147
VAL 148
VAL 149
MET 150
GLU 151
GLU 152
VAL 153
LYS 154

Residues[b] distinguishing all Global from all Local Lrps
ARG 14
LYS 25
ASP 26
ARG 28
ARG 54
TYR 69
SER 73
PHE 77
ASN 83
ALA 86
GLN 92
ILE 161
THR 163
ARG 164

Residues[c] acetylated in EcoLrp
LYS 25
LYS 36
LYS 129

a – Based on the 40 species included in this study, with ten each from the Enterobacteriales, Vibrionales, Alteromonadales and Pasteurellales. Numbering is from PDB 2GQQ (*E. coli* Lrp), and differs from numbering in the rest of the figures (which is based on the multiple alignment).

b – Assuming that all ten Enterobacteriales and Vibrionales Lrps used are global, all ten Pasteurellales are local, and excluding the Alteromonadales. This set derived from the order-specific Logos in Figure 3A.

c – Taken from supplementary information of reference 47.
